# Social mind at rest: resting-state connectivity associated with social thought relates to friendship quality

**DOI:** 10.1093/scan/nsag069

**Published:** 2026-08-25

**Authors:** Yi-Sheng Wong, Junhong Yu

**Affiliations:** Division of Mathematical Sciences, School of Physical and Mathematical Sciences, Nanyang Technological University, 637371, Singapore; Psychology, School of Social Sciences, Nanyang Technological University, 639818, Singapore; Yeo Boon Khim Mind Science Centre, National University of Singapore, 119228, Singapore

**Keywords:** mind wandering, thought content, content regulation hypothesis, spontaneous cognition

## Abstract

Although mind wandering is often associated with adverse outcomes, certain thought content may support adaptive functions. This study investigated how latent dimensions of resting-state thought relate to adaptive functioning, mental, emotional, and behavioral problems, and resting-state connectivity patterns in 130 healthy adults. Exploratory factor analysis of the New York Cognition Questionnaire identified seven thought dimensions: five content-related factors (Social Cognition, Self-Oriented Reflection, Interpersonal Reflection, Near-Term Past/Future, and Positive & Meaningful) and two form-related factors (Specificity and Imagery). This is partially consistent with prior work, especially for the social dimension. Controlling for sex and age, partial Pearson correlations showed that Social Cognition and Interpersonal Reflection were positively associated with friendship quality. Vertex-wise analyses identified multiple cortical clusters linked to distinct thought dimensions, and network-based statistics revealed that Social Cognition was associated with resting-state functional connectivity (rsFC) primarily between the default mode and ventral attention networks. Notably, both positive and negative rsFC network strengths related to Social Cognition were associated with friendship quality. These findings collectively support the content regulation hypothesis, suggesting that specific thought dimensions engage distinct neural networks and relate to meaningful social outcomes. Overall, the results highlight the importance of integrating subjective phenomenological experience with objective neural measures.

## Introduction

A remarkable feature of the human mind is its persistent tendency to drift away from the immediate sensory environment toward a stream of thoughts—a phenomenon known as mind wandering (Smallwood and Schooler 2006). Far from being a rare lapse in attention, research indicates that we spend a substantial portion of our waking life in this state (Kane *et al* 2007, Killingsworth and Gilbert 2010). Yet, despite its ubiquity, the functional significance of mind wandering remains puzzling. On one hand, a growing body of work highlights its considerable costs (for reviews, see Mooneyham and Schooler 2013, Smallwood and Schooler 2015, Zanesco *et al.* 2025). On the other hand, a wandering mind is not without its benefits (e.g. Girardeau *et al.* 2023, Simor *et al.* 2025, Vékony *et al.* 2025). Spontaneous thought is theorized to support several adaptive functions, such as facilitating future planning, enabling autobiographical reflection, and helping individuals refine long-term goals (Baars 2010, Mills *et al.* 2018, Mildner and Tamir 2024). This apparent duality raises a central question: how can a single cognitive state give rise to both costs and benefits?

The resolution to this paradox lies not in the mere occurrence of mind wandering but in the “content” of the thoughts that arise (Dane 2018, Ibaceta *et al.* 2024). For instance, Andrews-Hanna *et al.* (2010) found that when individuals engaged in self-relevant reflections on meaningful past or future events, components of the default mode network associated with mnemonic scene construction and the evaluation of personal significance were coactivated. This suggests an adaptive role for spontaneous thought in supporting future-oriented mental simulation and personally meaningful cognition. Similarly, episodes of mind wandering that center on social relationships have been associated with enhanced social-cognitive abilities (Poerio *et al.* 2017). These findings collectively support the content regulation hypothesis (Smallwood and Andrews-Hanna 2013), which proposes that the consequences of mind wandering are determined not simply by the diversion of attention from the task at hand, but by the nature and direction of the thoughts that capture attention (see also Andrews-Hanna *et al.* 2014, Smallwood and Schooler 2015).

One important tool for characterizing the content of thought is the New York Cognition Questionnaire (NYC-Q, Gorgolewski *et al.* 2014). In the original study, the researchers identified five dimensions of thought: social cognition, future-oriented, past-oriented, positive, and negative thought. Subsequent research using Multi-Dimensional Experience Sampling (MDES) has similarly shown that ongoing thought can be described along recurring dimensions, including social content, temporal focus, and emotional tone (e.g. Sormaz *et al.* 2018, Turnbull *et al.* 2019). Recent neuroscience research has also demonstrated that such dimensions of thought are not merely subjective epiphenomena but are systematically linked to patterns of brain activity and functional connectivity (e.g. Lux *et al.* 2022, Hardikar *et al.* 2024, Kim *et al.* 2024).

Indeed, the content and dynamics of thought are not only crucial to understanding its role in normative cognition but also provide a valuable lens for examining psychopathology. Atypical profiles of mind wandering are a well-documented feature of numerous psychiatric conditions (e.g. Bozhilova *et al.* 2018, Simpraga *et al.* 2021), with dysregulated patterns such as ruminative, perseverative, or negatively valenced thought cutting across traditional diagnostic categories including depression (Hoffmann *et al.* 2016), attention deficit hyperactivity disorder (ADHD; Lanier *et al.* 2021), and anxiety disorders (Fell *et al.* 2023). This perspective motivates a shift from categorical approaches toward more dimensional frameworks that investigate how specific features of thought map onto diverse behavioral and emotional outcomes (Christoff *et al.* 2016, Kucyi *et al.* 2023).

In line with this shift, resting-state functional magnetic resonance imaging (rs-fMRI) has been increasingly used to examine how resting-state regional homogeneity (ReHo, i.e. local synchronization of spontaneous brain activity, Zang *et al.* 2004) and functional connectivity (rsFC) relate to behavioral phenotypes, personality traits, and clinical symptoms (e.g. Altinok *et al.* 2021, Chen *et al.* 2022, Li *et al.* 2024, Wong *et al.* 2024, Wong and Yu, 2024). However, a substantial explanatory gap remains. Specifically, the interpretations of ReHo- and rsFC-behavior associations are often abstract and highly inferential, as the psychological processes unfolding during rest are seldom assessed directly (Smallwood and Schooler 2015, Yu *et al.* 2025).

Recent work has proposed that resting-state thought content may serve as a critical bridge between resting-state connectivity patterns and behavior. According to Yu *et al.* (2025), stable traits and behavioral dispositions shape the characteristic themes of individuals’ ongoing thoughts, which in turn influence connectivity patterns during rest. From this perspective, thought content is not an incidental by-product but a key mediating variable that provides essential psychological context for interpreting brain–behavior relationships (see also Hardikar *et al.* 2024). This conceptual shift underscores the need to move beyond simple correlational mapping toward more complete mechanistic models that link brain activity, thought content, and behavior outcomes (see also Kucyi *et al.* 2023).

Given the well-established role of thought content in shaping the functional consequences of mind wandering, its clear relevance to psychopathology, and the growing theoretical emphasis on integrating subjective experience into neuroimaging research, the present study investigated the interrelationships among mind wandering, adaptive functioning, mental, emotional, and behavioral problems, and resting-state connectivity patterns using data from 130 healthy adults. The study had four primary aims: (i) to characterize the latent factor structure of thought content and form during the resting-state scan using exploratory factor analysis of the NYC-Q (Gorgolewski *et al.* 2014), (ii) to evaluate how these empirically derived thought dimensions relate to adaptive functioning and mental, emotional, and behavioral problems as assessed by the Adult Self Report (ASR, Achenbach and Rescorla 2003), (iii) to identify the ReHo clusters and rsFC networks associated with each thought dimension, and (iv) to examine whether ReHo clusters and/or rsFC network strengths associated with thought dimensions were associated with self-reported adaptive functioning and mental, emotional, and behavioral problems. Building on prior NYC-Q and MDES work, the main contribution of the present study is the integration of empirically derived thought dimensions with adaptive functioning, ReHo, and rsFC measures in a single sample.

Based on previous studies (e.g. Gorgolewski *et al.* 2014, Sormaz *et al.* 2018, Turnbull *et al.* 2019), we expected the factor solution to recover dimensions related to social thought as well as temporal and affective dimensions. Drawing on the content regulation hypothesis (Smallwood and Andrews-Hanna 2013) and evidence that social thought supports social-cognitive abilities (Poerio *et al.* 2017), we further hypothesized that socially oriented thought dimensions would be positively associated with adaptive social functioning. Given the established roles of the default mode network in social cognition and self-referential processing (e.g. Buckner *et al.* 2008, Andrews-Hanna *et al.* 2014), we anticipated that social thought dimensions would be associated with rsFC patterns involving the default mode network. We also expected ReHo associations to involve regions implicated in mentalizing such as the medial prefrontal cortex (Amodio and Frith 2006).

## Materials and methods

### Participants

This study analyzed data from 130 native German-speaking adults (55 females and 75 males) between the ages of 20 and 60. The original public release (Mendes *et al.* 2019) contained a broad set of phenotypic assessments from 204 healthy participants. For the current analysis, 74 participants were excluded due to the following reasons: not completing the NYC-Q (*n *= 44), not completing the ASR (*n *= 6), not completing both the NYC-Q and the ASR (*n *= 3), error in data entry in the NYC-Q (*n *= 1, subject had an invalid score of 17 recorded for Q12), not having any valid fMRI runs [i.e. either missing or excessive head motion during scanning (root mean squared displacement > 0.25), *n *= 1], and being aged 60 years and above (*n *= 19). Participants aged over 60 years were excluded because the ASR is validated only for use in adults aged 18–59 years (Achenbach and Rescorla 2003). A post-hoc sensitivity power analysis using G*Power version 3.1.9.7 (Faul *et al.* 2009) indicated that the final sample size of 130 is sufficient to detect a small-to-medium effect size of *r* = 0.24 for correlation analyses, given power of 80% and α of 0.05 (two-tailed).

Due to anonymity concerns, participants’ ages were grouped into 5-year intervals, and the median 5-year age bin was between 25 and 30. For ease of analysis, both participants’ sexes (female = 1, male = 2) and the 5-year age bins (e.g. 20 − 25 = 1, 25 − 30 = 2) were recoded into integers. All participants provided written informed consent prior to participation and received monetary compensation for their participation. The study received ethical approval from the University of Leipzig Human Ethics Committee (097/15-ff).

### Questionnaires

All instructions and questions/questionnaires were presented in German.

#### New York Cognition Questionnaire

The NYC-Q (Gorgolewski *et al.* 2014) is a 31-item scale designed to measure the content and form of thoughts experienced while performing a particular activity. It contains two sections. The first section consists of 23 items measuring the content of thoughts. Participants responded to items such as “I thought about an event that took place earlier today” on a 9-point Likert scale, ranging from 1 (“completely did not describe my thoughts”) to 9 (“completely did describe my thoughts”). The second section consists of eight items assessing the form of thoughts. Participants responded to items such as “My thoughts were in the form of words” on a 9-point Likert scale, ranging from 1 (“completely does not characterize my experience”) to 9 (“completely does characterize my experience”). Immediately after completion of the scanning session, participants were asked to indicate the extent to which each of these items correctly characterizes their thinking. Note that according to Mendes *et al.* (2019), Cronbach’s α was not computed for the NYC-Q, as the questionnaire consists of heterogeneous items that do not reflect a single unitary construct and thus is not intended to demonstrate internal consistency.

#### Adult Self Report

The ASR (Achenbach and Rescorla, 2003) is a 162-item scale for adults (ages 18–59) that measures adaptive functioning and mental, emotional, and behavioral problems experienced in the past 6 months. It contains two sections. The first section consists of 36 items measuring adaptive functioning domains. Participants responded to items describing the quantity and quality of relationships, education level, and job satisfaction on either a 3-point or 4-point Likert scale. The second section consists of 126 items assessing a broad range of problem areas, including psychological syndromes, DSM-oriented problems, and substance use. Participants responded to items such as “I have trouble planning for the future” on a 3-point Likert scale, ranging from 0 (“not true”) to 2 (“very true or often true”). Individual item scores within each subscale were summed to generate composite syndrome scale scores, providing an overall index of the participant’s level of problems within each domain.

According to Mendes *et al.* (2019), two items were erroneously omitted from their dataset, which affected scoring for the Somatic Complaints subscale and the broader Internalizing composite scale. In addition, responses to the ASR’s open-ended questions could not be examined, as Mendes *et al.* (2019) did not make those narrative comments publicly available. Finally, we did not analyze three adaptive functioning items related to spouse/partner relationships, employment, and education due to high rates of missing data. The adaptive functioning domains retained for analysis are presented in Table 1.

**Table 1:** Partial Pearson correlations among the New York Cognition Questionnaire factors and the Adult Self Report domain scores after controlling for sex and age.

	1.	2.	3.	4.	5.	6.	7.	8.	9.	10.	11.	12.	13.	14.	15.	16.	17.	18.	19.	20.	21.	22.
1.	—																					
2.	.04	—																				
3.	**.25**	**.17**	—																			
4.	.15	**–.22**	**.19**	—																		
5.	**.26**	.15	.12	.09	—																	
6.	**.19**	**.19**	.02	–.05	.12	—																
7.	.14	**.25**	**.25**	–.08	.08	.07	—															
8.	**.27**	.05	**.29**	.01	**.22**	.00	.17	—														
9.	–.14	–.17	–.09	–.05	.03	.07	–.11	.07	—													
10.	–.10	–.09	.00	–.02	–.06	–.12	–.10	–.02	–.09	—												
11.	.13	.00	.12	.11	.08	–.09	.10	***.32***	–.09	**.24**	—											
12.	.04	–.04	.08	.06	–.08	.00	–.08	**.21**	.00	**.28**	**.25**	—										
13.	.01	–.04	.08	.06	–.09	–.02	.12	–.13	**–.27**	**.25**	**.19**	**.28**	—									
14.	–.09	–.13	.12	.17	–.09	–.13	.00	**–.26**	–.16	**.20**	.06	.11	***.73***	—								
15.	**–.23**	–.07	–.05	–.03	**–.20**	–.09	–.06	***–.41***	**–.20**	**.21**	–.03	–.07	***.54***	***.70***	—							
16.	.13	–.06	.05	.03	–.03	–.04	–.07	–.09	–.09	**.17**	.05	**.24**	***.41***	***.40***	**.26**	—						
17.	.02	–.01	.05	.00	–.13	.17	.07	–.10	–.13	**.18**	.05	.16	***.75***	***.50***	***.43***	***.39***	—					
18.	–.10	–.13	.16	.07	–.03	**–.19**	.02	–.11	–.12	**.24**	.14	.16	***.64***	***.68***	***.43***	***.36***	***.39***	—				
19.	–.08	–.01	.06	.11	.01	–.01	.01	**–.25**	**–.20**	.16	.08	.14	***.62***	***.64***	***.47***	***.44***	***.34***	***.49***	—			
20.	.00	–.02	.10	.09	.03	–.09	.00	.01	–.14	***.36***	***.43***	***.29***	***.59***	***.47***	***.36***	**.20**	***.37***	***.51***	***.41***	—		
21.	.07	.05	**.23**	.14	.08	–.01	**.20**	–.04	**–.17**	**.27**	***.30***	.10	***.51***	***.35***	.16	**.28**	***.30***	***.39***	***.54***	***.48***	—	
22.	–.10	–.11	.07	.10	–.13	–.12	–.03	***–.32***	**–.19**	**.23**	.04	.10	***.73***	***.95***	***.84***	***.56***	***.54***	***.64***	***.66***	***.46***	***.34***	—
23.	–.02	.00	.14	.14	.04	–.05	.06	–.15	**–.22**	***.30***	***.29***	**.22**	***.72***	***.64***	***.45***	***.40***	***.42***	***.58***	***.88***	***.75***	***.77***	***.64***

*N *= 130. Variables: (1) NYC-Q-Social Cognition; (2) NYC-Q-Self-Oriented Reflection; (3) NYC-Q-Interpersonal Reflection; (4) NYC-Q-Near-Term Past/Future; (5) NYC-Q-Positive & Meaningful; (6) NYC-Q-Specificity; (7) NYC-Q-Imagery; (8) ASR-Friends; (9) ASR-Family; (10) ASR-Tobacco/times per day; (11) ASR-Alcohol/days drunk in the past six months; (12) ASR-Drugs/days used in the past six months; (13) ASR--Critical Items; (14) ASR-Anxious Depressed; (15) ASR-Withdrawn; (16) ASR-Somatic Complaints; (17) ASR-Thought Problems; (18) ASR-Attention Problems; (19) ASR-Aggressive Behavior; (20) ASR-Rule-Breaking Behavior; (21) ASR-Intrusive Behavior; (22) ASR-Internalizing; (23) ASR-Externalizing.

Formatting denotes significance: bold = *P* < .05; bold + underline = *P* < .01; bold + underline + italic = *P* < .001.

### Magnetic resonance imaging data acquisition

The magnetic resonance imaging (MRI) data were recorded on a 3 T scanner (MAGNETOM Verio, Siemens Healthcare, Erlangen, Germany) equipped with a 32-channel head coil. The high-resolution structural image was recorded using a 3D MP2RAGE sequence (Marques *et al.* 2010). For the parameters, see Supplementary material.

Four rs-fMRI scans were obtained in axial orientation using T2*-weighted gradient-echo echo-planar imaging with multiband acceleration. The phase-encoding direction was A-P for runs 1 and 3, and P-A for runs 2 and 4. During the scans, participants were instructed to remain awake and lie still with their eyes open while looking at a fixation cross. For the parameters, see Supplementary material.

### Resting-state functional MRI data preprocessing

The rs-fMRI volumes were preprocessed using fMRIPrep version 24.0.1 (Esteban *et al.* 2019) using default parameters. For more details, see Supplementary material.

### Statistical analyses

Analyses were performed using R version 4.5.2 (R Core Team, 2025), RStudio version 2025.09.2 (Posit Team, 2025), and JASP version 0.95.4 (JASP Team, 2025). An α of 0.05 was chosen to signify statistical significance for all analyses. Benchmarks of 0.10, 0.30, and 0.50 were used to indicate small, moderate, and large effect sizes (Cohen 1988).

#### Behavioral analysis

Using the R code (https://github.com/NeuroanatomyAndConnectivity/NYC-Q) provided by Gorgolewski *et al.* (2014), we conducted exploratory factor analysis to decompose the NYC-Q items into latent factors. In line with prior work, the two sections of the NYC-Q (content and form of thoughts) were analyzed separately to identify interpretable latent dimensions. The number of factors to retain was determined using parallel analysis (Horn 1965) with 1000 iterations. Factors were then extracted via principal axis factoring with oblimin rotation (Revelle 2025), which allows for correlated latent constructs and improves interpretability. Item-level responses were subsequently transformed into factor scores using the refined method of ten Berge *et al.* (1999), which preserves the correlation structure between latent variables. Additional preprocessing steps, including the generation of composite regressors and merging with relevant phenotypic measures, were carried out as described in the original pipeline (Gorgolewski *et al*. 2014) to ensure comparability and reproducibility.

Partial Pearson correlations were used to examine associations between the NYC-Q factor scores and ASR domain scores while controlling for sex and age. These covariates, which were treated as categorical controls, were included due to consistent evidence of sex- and age-related influences on resting-state connectivity patterns (e.g. Foo *et al.* 2021, Montalà-Flaquer *et al.* 2023, Al Zoubi *et al.* 2022). Bonferroni correction was applied to account for multiple comparisons. We defined a hypothesis-driven primary correction family comprising the correlations between the retained NYC-Q factors and the two ASR adaptive-functioning domains: Friends and Family. This primary family was selected because the study specifically hypothesized that socially oriented thought would be associated with adaptive social functioning. We also report on whether the primary findings remained significant under a more conservative Bonferroni correction across all cross-domain NYC-Q–ASR correlations.

#### Resting-state regional homogeneity analysis

Vertex-wise analyses were performed using the R package “VertexWiseR” version 1.4.5 (Billaud and Yu, 2024) to identify ReHo clusters significantly associated with NYC-Q factors, controlling for sex and age. ReHo provides a reliable and sensitive measure of local neural synchrony, reflecting the temporal coordination of activity among neighboring brain regions by quantifying the similarity of each voxel’s time series with those of its immediate neighbors (Zang *et al.* 2004). Depending on the functional and structural context of a region, lower regional ReHo values in a condition typically indicate reduced regional specialization, increased functional flexibility, or impaired function within that local network (Jiang and Zuo 2016).

Whole-brain vertex-wise statistics were thresholded at *P* < .05 using a cluster-wise correction based on random field theory (RFT, Worsley *et al.* 1999, Larivière *et al.* 2023). RFT is well suited to this application because it explicitly models the spatial smoothness and structure of brain data, thereby reducing the risk of inflated false positives that may arise when voxels are treated as independent observations, while also improving sensitivity (Friston *et al.* 1994, Worsley *et al.* 1996). In contrast to false discovery rate procedures, which do not account for spatial dependence, RFT leverages spatial correlations to provide more accurate cluster-level inferences (Lindquist and Mejia 2015). Moreover, compared with other family-wise error rate controls such as Bonferroni correction, RFT offers a less conservative yet still robust control of Type I error, yielding a closer approximation to the true false positive rate (Lindquist and Mejia 2015). For cluster identification, we used the Desikan atlas (Desikan *et al.* 2006), a widely adopted cortical parcellation scheme that balances anatomical interpretability with spatial resolution and facilitates comparability across neuroimaging studies. Significant ReHo clusters were subsequently entered into regression analyses.

#### Resting-state functional connectivity analysis

Network-based statistics (Zalesky *et al.* 2010), as implemented in the R package “NBR” version 0.1.5 (Gracia-Tabuenca and Alcauter, 2020), were used to identify networks of resting-state functional connectivity (rsFC) edges significantly associated with NYC-Q factors, controlling for sex and age. This method is a data-driven/model-free approach that controls for the family-wise error rate associated with mass univariate testing typical of whole-brain analysis (Zalesky *et al.* 2010). At the initial stage, individual edges in the functional connectome (i.e. interrelated nodes) were analyzed using the *t*-statistic. Edges were filtered out if they were not significantly associated with NYC-Q factors. Next, under the assumption that neighboring edges are usually anatomically related (Bullmore and Sporns 2009), networks were formed using the significant edges parsed at the previous step. Selection thresholds were set at *P* < 0.001 and *P* < 0.05 at the edge and network levels, respectively. Statistical significance at the network level was determined by testing its network strength against a null distribution generated by 1000 nonparametric permutations. For each of the NYC-Q factors, the significant edges that survived both levels of thresholds were grouped into positive and negative networks, corresponding to their positive and negative associations with the task variable. Then, the positive and negative network strengths were calculated by summing up these positive and negative edges in their respective groups. These network strengths were then used in subsequent regression analyses.

#### Regression analysis

Using regression analyses, we investigated whether the ReHo clusters and rsFC network strengths associated with each thought dimension were associated with ASR domain scores, controlling for sex and age. Note that these regression analyses were interpreted as associative tests rather than out-of-sample predictive models.

## Results

### Questionnaires

**Figure 1:**
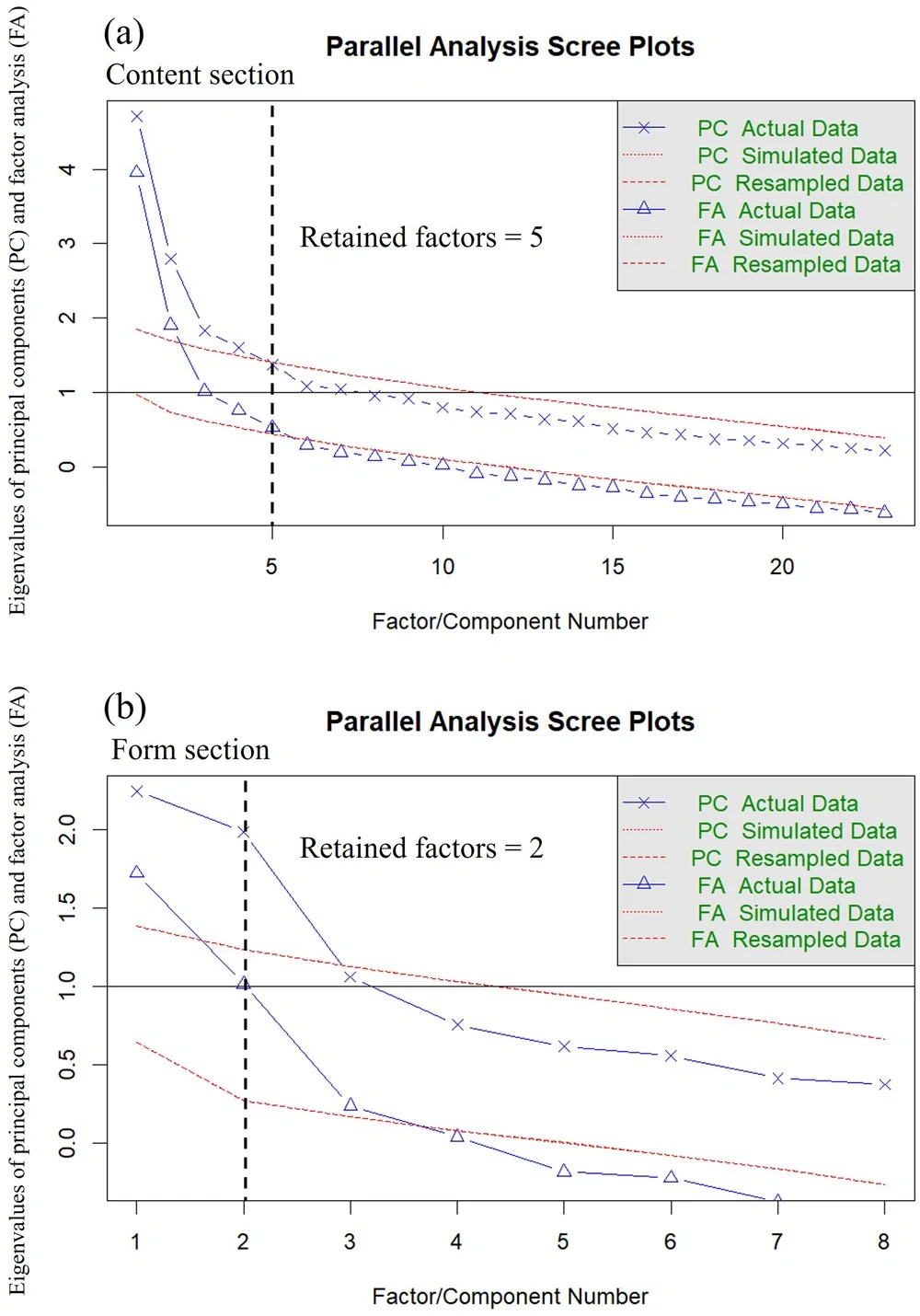
Parallel analysis scree plots. (a) The content section of the NYC-Q and (b) the form section of the NYC-Q. The vertical reference lines mark the retained number of factors for each section (five for content and two for form). Principal component and factor analysis solutions are shown for actual, simulated, and resampled data.

Parallel analysis revealed a seven-factor solution underlying the NYC-Q, with five factors emerging for the first section (see Fig. 1a) and two factors emerging for the second (see Fig. 1b).

**Figure 2:**
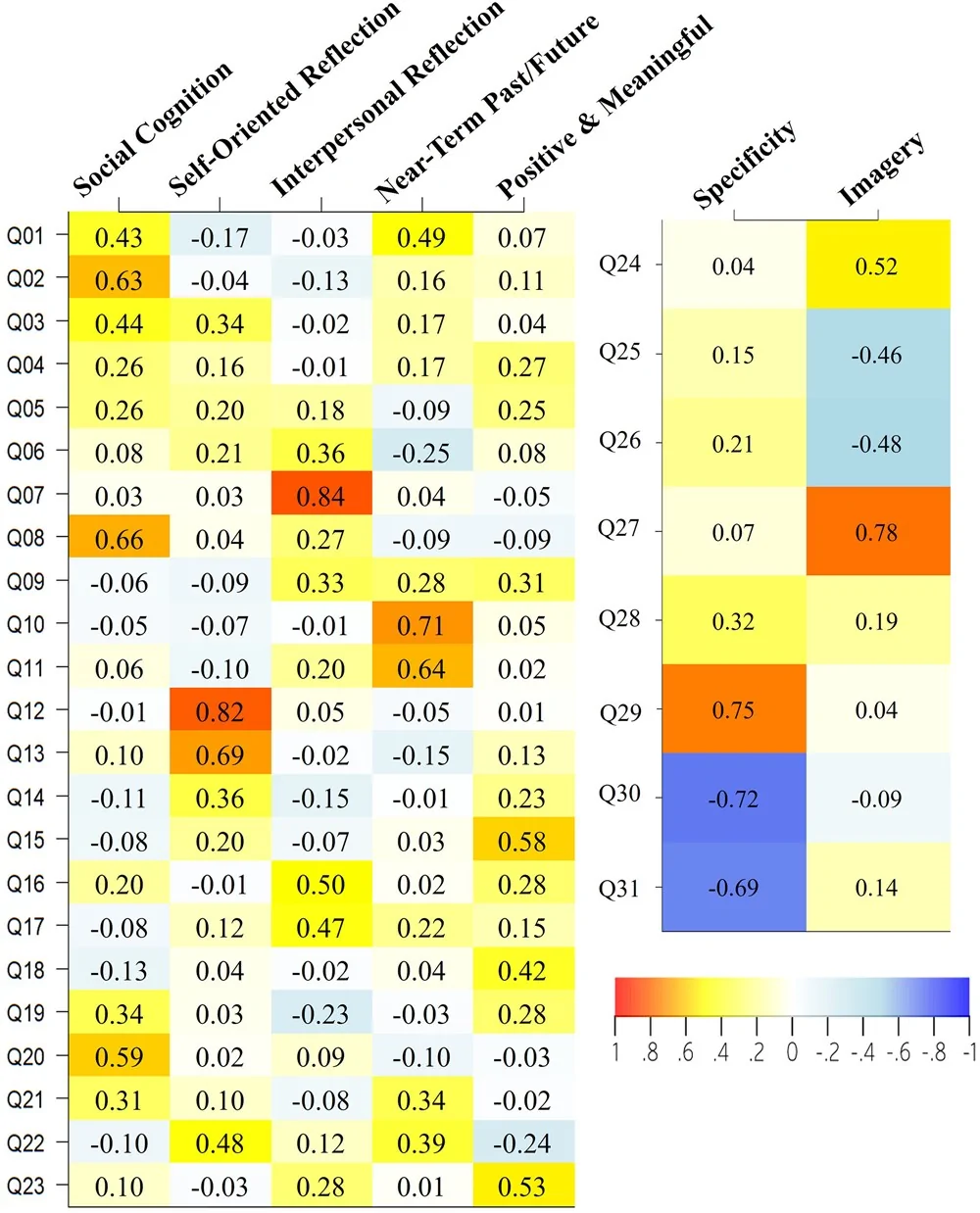
Factor loadings on the items from the NYC-Q describing the content (left panel; Q01–Q23) and form of thoughts (right panel; Q24–Q31). *Note. N* = 130. Questions (rows) were decomposed into factors (columns) using exploratory factor analysis. Similar to Gorgolewski *et al*. (2014), factors were named based on subjective interpretation of the loadings. Weights (i.e. how much each item contributes to each factor) are represented both numerically as well as on a color scale.

For the first section of the NYC-Q, exploratory factor analysis demonstrated good fit to the data [root mean square error of approximation (RMSEA) < 0.05, Brown 2015], *χ*²(148) = 190.09, *P* = 0.011, RMSEA = 0.046 [90% CI (0.024, 0.065)], root mean square of the residuals (RMSR) = 0.05, Tucker Lewis Index (TLI) = 0.885, and Bayesian Information Criterion (BIC) = –530.31. The five extracted factors (PA1-PA5) accounted for 42% of the total variance, with each factor contributing between 7% and 10%. Several items showed strong primary loadings (e.g. Q07 on Factor 3/PA3, *λ* = .84, Q12 on Factor 2/PA2, *λ* = .82, Q10 on Factor 4/PA5, *λ* = .71, see Fig. 2), and most items exceeded the conventional loading threshold of .40 (Hair *et al.* 2019). However, a few items exhibited weaker communalities (e.g. Q18, *h*² = .17), suggesting limited contribution to the factor structure. Factor intercorrelations were modest (*r* = –.18 to .28), indicating that the factors were related yet distinct. Measures of factor score adequacy (factor-score correlations = .83–.91, *R*² = .69–.83) supported the robustness and reliability of the five-factor solution.

**Figure 3:**
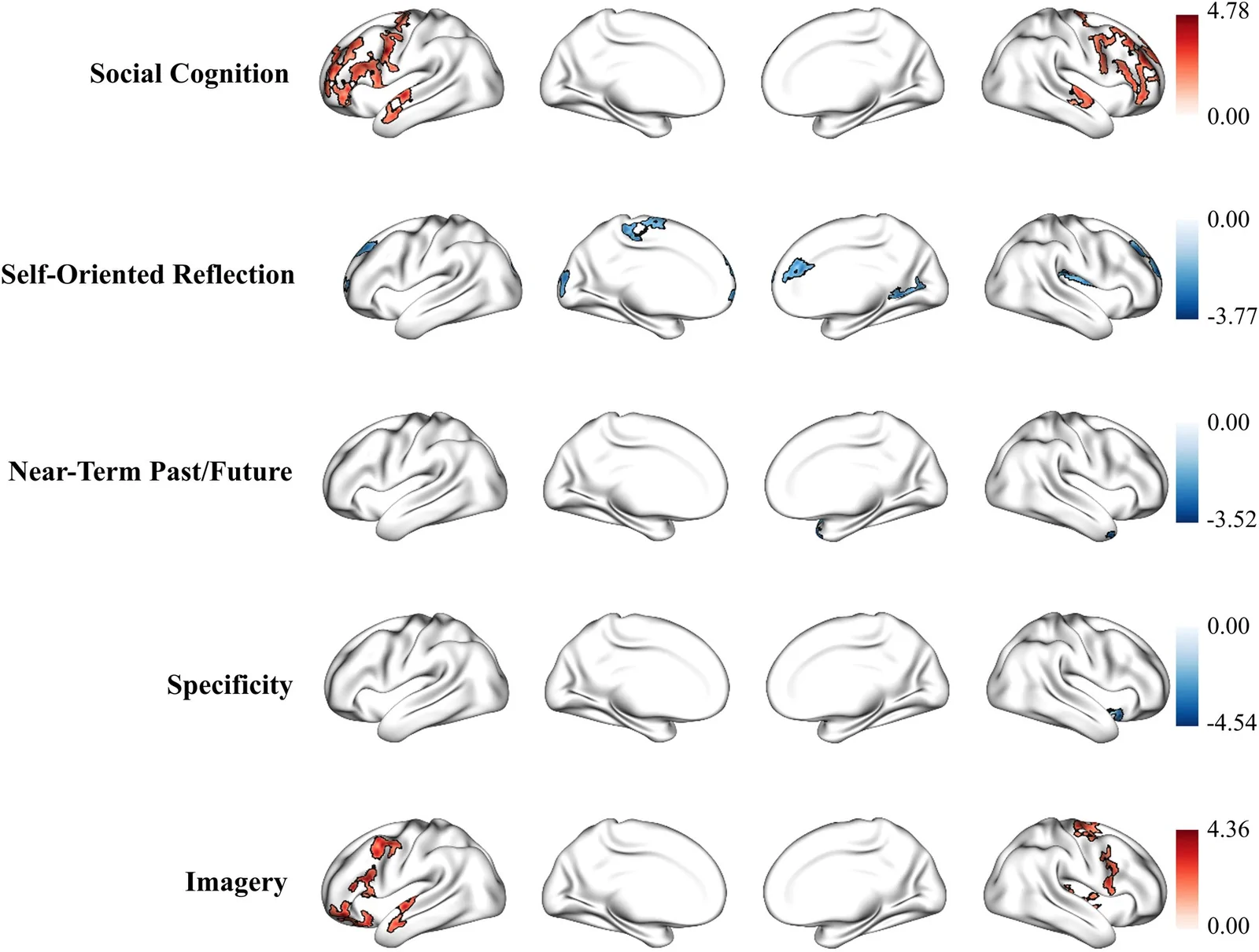
Regional homogeneity clusters associated with factors extracted from the New York Cognition Questionnaire. *Note*. *N* = 130. ReHo clusters showing positive associations (i.e. higher local synchrony) with New York Cognition Questionnaire factors are shown in red; clusters showing negative associations (i.e. lower local synchrony) are shown in blue.

We labelled these five factors as thoughts about close social relationships (“Social Cognition”), thoughts about self (“Self-Oriented Reflection”), thoughts about others and relationships (“Interpersonal Reflection”), thoughts about the near-term past and future (“Near-Term Past/Future”), and positive or meaningful thoughts (“Positive and Meaningful”). Social Cognition was characterized mainly by items concerning close or personally significant social bonds (e.g. Q02 and Q03), Self-Oriented Reflection by items concerning the self, personal worries, and self-relevant emotional experiences (e.g. Q13, Q14, and Q22), Interpersonal Reflection by items reflecting a broader and less relationship-specific form of thinking about others (e.g. Q06 and Q07), Near-Term Past/Future by items concerning temporally proximal events (e.g. Q10 and Q11), and Positive and Meaningful by items reflecting positive, cheerful, or personally meaningful thought content (e.g. Q15 and Q18). This five-factor content solution showed only limited convergence with the original NYC-Q content structure reported by Gorgolewski *et al.* (2014), which identified separate social-, past-, future-, positive-, and negative-thought factors, and prior MDES studies, which have identified dimensions related to social content, temporal focus, and emotional tone (e.g. Sormaz *et al.* 2018, Turnbull *et al.* 2019). The clearest overlap was observed for the social dimension. Beyond this, the correspondence was modest. Such differences, however, are not unexpected because exploratory factor solutions depend on the sample.

In contrast, the second section of the NYC-Q was best represented by a two-factor model (PA1 and PA2) identified through parallel analysis. Model fit was weaker than that of the first section, *χ*²(13) = 27.21, *P* = 0.012, RMSEA = 0.091 [90% CI (0.042, 0.141)], RMSR = 0.06, TLI = 0.831, and BIC = –36.07. Notably, the elevated RMSEA indicates only moderate model fit, suggesting that the two-factor structure should be interpreted with caution. The two factors explained 39% of the variance, with primary loadings observed for items such as Q27 (*λ* = .78) and Q29 (*λ* = .75, see Fig. 2). While most items loaded above .45, some items showed weaker communalities (e.g. Q28, *h*² = .14). Measures of factor score adequacy were acceptable (factor-score correlations = .85–.89, *R*² = .73–.78). We labelled these two factors as the specificity of thought (“Specificity”), and imagery-related thoughts (“Imagery”).

Next, we conducted partial Pearson correlations to examine the relationships among NYC-Q factor scores and ASR domain scores while controlling for sex and age (see Table 1). Within the primary adaptive-functioning family (seven NYC-Q factors × two retained ASR adaptive-functioning domains, Bonferroni-adjusted α = .0036), positive correlations were observed between NYC-Q-Social Cognition and ASR-Friends, *r* = .27, *P* = 0.0018, and between NYC-Q-Interpersonal Reflection and ASR-Friends, *r* = .29, *P* = 0.0011. No other NYC-Q-ASR adaptive-functioning correlation reached this corrected threshold. When applying a more conservative Bonferroni correction across the full exploratory set of 112 NYC-Q-ASR cross-measure correlations (adjusted α = .00045), no NYC-Q–ASR association remained significant.

Overall, these results suggest that, after accounting for sex and age, individuals with higher scores on the NYC-Q-Social Cognition and NYC-Q-Interpersonal Reflection tended to report greater friendship quality.

### Resting-state regional homogeneity

Vertex-wise analyses identified multiple cortical clusters that showed significant associations with resting-state thought dimensions after controlling for sex and age. ReHo patterns were found for five of the seven thought factors extracted from the NYC-Q (see Table 2 and Fig. 3). Overall, these results reveal distinct and dissociable ReHo patterns associated with different dimensions of thought.

**Figure 4:**
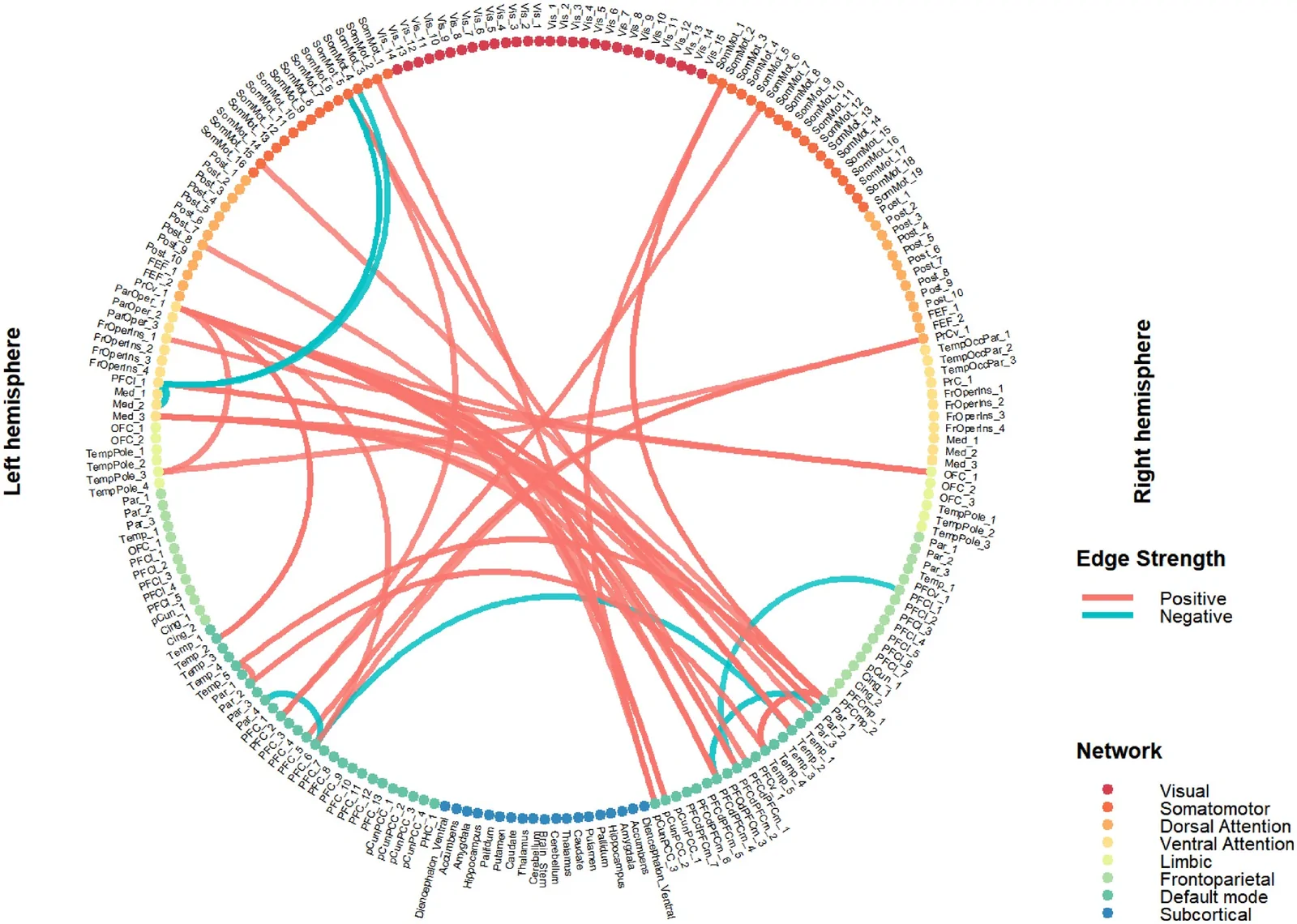
Connectogram showing the networks of resting-state functional connectivity edges related to NYC-Q—Social Cognition.

**Table 2:** Cluster level results from vertex-wise analyses.

Cluster ID	Number of vertices	*P* value	X	Y	Z	*t* stat	Region	Cluster ID	Number of vertices	*P* value	X	Y	Z	*t* stat	Region
Positive contrast associated with Social Cognition								Negative contrast associated with Self-Oriented Reflection							
1	1227	< .001	–35.9	49.1	20.8	4.02	Left rostral middle frontal	1	365	< .001	–17.5	37.4	41.2	–3.32	Left superior frontal
2	912	< .001	41.9	39.6	28.2	3.79	Right rostral middle frontal	2	282	.001	23.7	55.9	21.2	–3.77	Right rostral middle frontal
3	465	< .001	–60.9	–11.4	–2.6	4.28	Left superior temporal	3	296	.001	–5.5	–32.7	50.4	–2.72	Left paracentral
4	313	< .001	–52.2	3.3	36.2	4.78	Left precentral	4	220	.022	39.7	–26.6	21.2	–3.18	Right supramarginal
5	303	.001	–25.6	–13.3	55.6	3.42	Left precentral	5	183	.034	8.3	33.4	23.4	–2.80	Right caudal anterior cingulate
6	191	.008	55.2	30.6	6.7	3.15	Right pars triangularis	6	231	.035	20.7	–46.8	0.6	–3.27	Right lingual
7	219	.012	17.8	54.8	27.2	3.13	Right rostral middle frontal	7	193	.037	–14.2	–100.4	10.8	–3.11	Left lateral occipital
8	215	.024	52.5	–15.7	5.8	3.55	Right transverse temporal								
9	246	.027	30.6	–13.8	63.0	3.12	Right precentral								
Positive contrast associated with Imagery								Negative contrast associated with Near-Term Past/Future							
1	445	< .001	–44.4	42.1	–15.6	4.36	Left pars orbitalis	1	202	< .001	43.1	13.6	–39.8	–3.52	Right middle temporal
2	365	< .001	–59.5	–10.6	–3.4	3.71	Left superior temporal								
3	442	< .001	18.5	–3.0	68.7	3.58	Right superior frontal	Negative contrast associated with Specificity							
4	289	< .001	–51.2	17.1	24.4	4.09	Left pars opercularis	1	131	.041	30.4	17.3	–12.0	–4.54	Right insula
5	275	< .001	–37.9	4.8	45.6	4.08	Left caudal middle frontal								
6	225	.01	49.3	6.6	17.8	3.23	Right precentral								
7	172	.023	43.4	–13.7	–5.8	2.80	Right superior temporal								

*N *= 130. The MNI coordinates (i.e. X, Y, Z) indicate the location of the cluster peak. The region labels are identified by mapping the cluster peaks onto the Desikan atlas.

### Resting-state functional connectivity

Using network-based statistics, we found that NYC-Q-Social Cognition was linked to a combination of 40 positive and negative edges from a wide range of brain regions, *P* = 0.041 (see Fig. 4). In particular, we observed that the rsFC between the default mode network and the ventral attention network was positively associated with NYC-Q-Social Cognition. There were no networks that were significantly associated with other factors of the NYC-Q (all *P* > 0.05).

**Figure 5:**
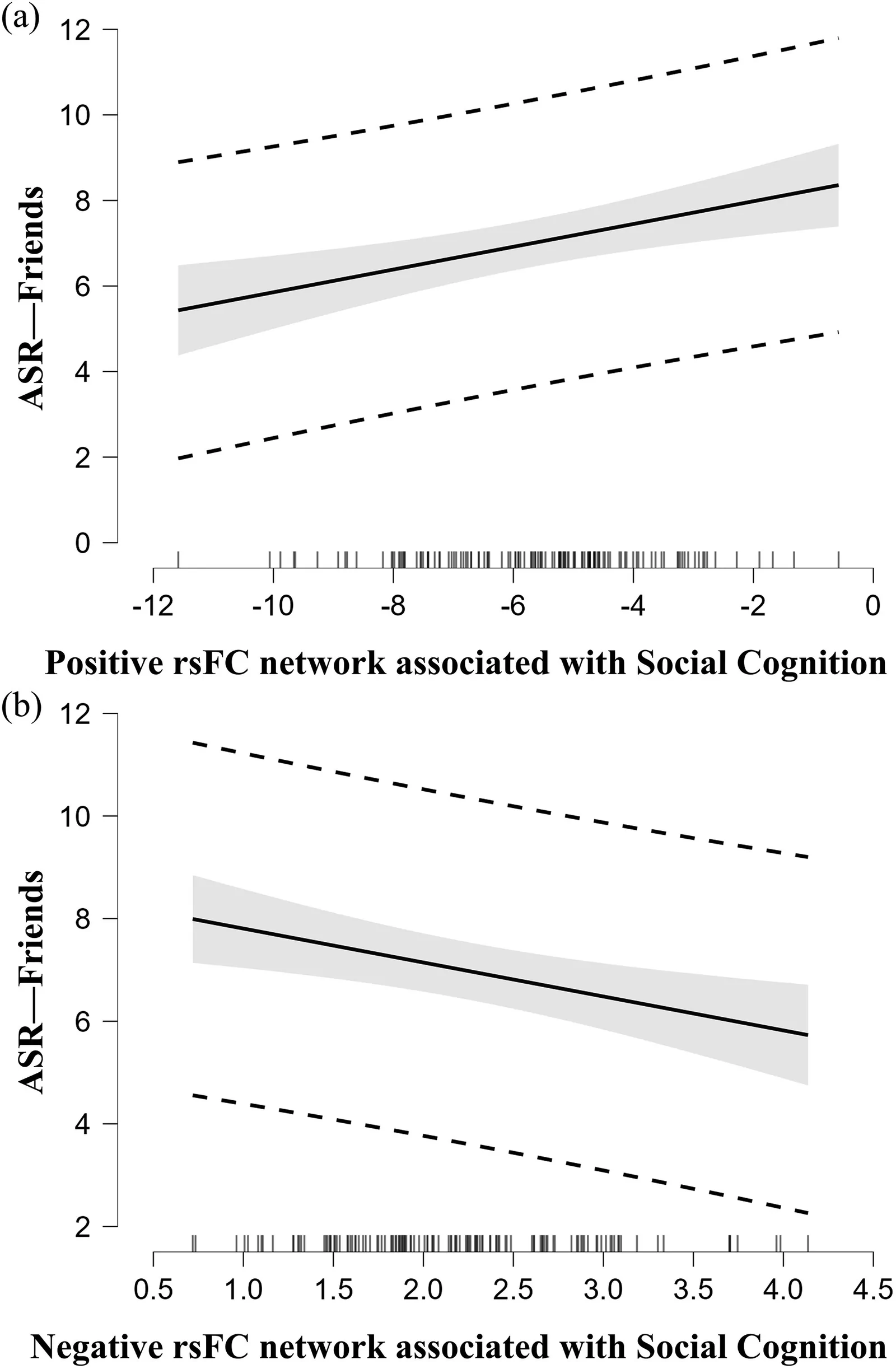
Marginal effects of the positive and negative rsFC networks associated with NYC-Q*—*Social Cognition on ASR*—*Friends. *Note*. *N* = 130. The solid line represents the marginal effects, and the dashed lines represent the 95% confidence intervals of model-estimated values.

### Resting-state connectivity patterns associated with friendship quality

Having identified ReHo clusters and rsFC networks associated with NYC-Q factors, we next investigated whether these connectivity patterns were associated with ASR domain scores. Because NYC-Q—Social Cognition was the only factor that showed significant associations with both ReHo and rsFC (see Table 2, Figs. 3 and 4), we used its corresponding resting-state connectivity patterns as the independent variables. Likewise, ASR-Friends was selected as the outcome variable because it was the adaptive-functioning domain that exhibited an association with NYC-Q-Social Cognition (see Table 1).

A significant association emerged between positive rsFC network strength and ASR—Friends scores. Specifically, after controlling for sex and age, stronger positive network strength was associated with higher ASR-Friends scores, standardized coefficient = 0.28, SE = 0.08, *t*(120) = 3.44, *P* = .0008, *R*^2^ = 0.23 (see Fig. 5a). A weaker negative network strength was also associated with higher ASR—Friends scores, standardized coefficient = −0.26, *SE *= 0.21, *t*(120) = −3.08, *P* = .0026, *R*^2^ = 0.21 (see Fig. 5b). No significant associations were found for ReHo cluster strength, standardized coefficient = 0.12, SE = 6.37, *t*(120) = 1.46, *P* = .147, *R*^2^ = 0.16.

## Discussion

The present study investigated how latent dimensions of thought content and form during rest relate to adaptive functioning and mental, emotional, and behavioral problems, as well as to resting-state connectivity patterns. Using an integrated multimethod approach that combined behavioral analysis, local and network-based connectivity mapping, and regression analysis on data from 130 healthy adults, we revealed: (i) seven dimensions of thought, comprising five content-related and two form-related factors that emerged from the exploratory factor analysis, (ii) significant positive correlations between the NYC-Q-Social Cognition and ASR-Friends scores, as well as between NYC-Q-Interpersonal Reflection and ASR-Friends scores, (iii) multiple cortical ReHo clusters that showed significant associations with distinct resting-state thought dimensions, (iv) significant associations between NYC-Q-Social Cognition and rsFC involving the default mode and ventral attention networks, and (v) both positive and negative rsFC network strengths related to Social Cognition were associated with friendship quality. Thus, the results were somewhat expected. A social thought dimension emerged clearly and this was highly consistent with prior work using the NYC-Q and MDES. However, temporal and affective content did not reproduce the separable structure reported in the original NYC-Q study (Gorgolewski *et al.* 2014). Nevertheless, the findings extend previous research by relating empirically derived dimensions of resting-state thought to friendship quality and to both local and network-level resting-state connectivity patterns.

A central theoretical framework motivating this study is the content regulation hypothesis (Smallwood and Andrews-Hanna 2013). The current findings provided further support for this hypothesis, showing that specific dimensions of social thought content were associated with friendship quality (see Table 1). Moreover, the positive correlations observed between NYC-Q-Social Cognition and NYC-Q-Interpersonal Reflection with friendship quality are aligned with prior research (Poerio *et al.* 2017). Although both Social Cognition and Interpersonal Reflection were associated with friendship quality, it is important to clarify the conceptual distinction between these factors. As noted in Results section, Social Cognition captures thoughts about close, personally significant social bonds, whereas Interpersonal Reflection reflects a broader form of thinking about others not restricted to close ties. Their modest correlation (*r* = .25) indicates partial overlap but also separability. Furthermore, only Social Cognition showed significant rsFC patterns involving default mode–ventral attention network interactions. This selectivity arises potentially because thoughts about close relationships likely involve more vivid, personally relevant social simulation, which may require coordinated recruitment of internally oriented mentalizing and self-referential processes supported by the default mode network (e.g. Buckner *et al.* 2008, Andrews-Hanna *et al.* 2014), together with externally oriented attentional reorienting mechanisms associated with the ventral attention network (Corbetta *et al.* 2008). By contrast, interpersonal reflection may rely on more diffuse neural substrates that were not captured at the network level using the current thresholds.

Overall, these results suggest that individuals who reported more frequent thoughts about close relationships and interpersonal dynamics also tended to report higher-quality friendships, consistent with the finding that mental simulation of social scenarios is linked to the maintenance and refinement of social competencies (Petzold *et al.* 2023, Shin *et al.* 2025). However, the opposite direction is also plausible: individuals with higher-quality friendships may encounter richer and more frequent social information, which in turn provides more content for social-cognitive processing during rest. This possibility is consistent with interpersonal models emphasizing that close relationships are maintained through repeated social interaction, responsiveness, and self-disclosure (Laurenceau *et al.* 1998). Notably, none of the other extracted thought dimensions showed significant associations with friendship quality after applying Bonferroni correction (see Table 1). This specificity in the thought–friendship relationship indicates that the content and directedness of thought toward social and relational themes constitute a more selective correlate of social outcomes.

The most striking finding from the ReHo analyses was the robust and spatially distributed pattern of elevated local synchrony associated with Social Cognition (see Table 2 and Fig. 3). Specifically, Social Cognition was linked to increased ReHo across nine cortical clusters encompassing key social-cognitive and motor regions, with the two largest clusters located bilaterally in the rostral middle frontal cortex. This region has been consistently identified as a core hub of the mentalizing network in task-based fMRI studies investigating theory of mind, perspective-taking, and mental states inference (e.g. Amodio and Frith 2006, Fehlbaum *et al.* 2022, Gan *et al.* 2024, Geisler and Meyer 2025). Relatedly, studies have shown that representations of self-relevant and social content can be decoded from patterns of spontaneous thought (e.g. Lux *et al.* 2022, Hardikar *et al.* 2024, Kim *et al.* 2024), further supporting the view that resting-state neural activity reflects meaningful social-cognitive processing (Poerio *et al.* 2017).

Extending prior work that has largely focused on the role of the default mode network in mind wandering (e.g. Kucyi *et al.* 2016, Wen *et al.* 2020, Zhang *et al.* 2022), we identified specific rsFC networks associated with social and interpersonal thought dimensions (see Fig. 4). The rsFC pattern associated with Social Cognition—marked primarily by positive connectivity between the default mode and ventral attention networks—is particularly noteworthy. A large body of research shows that the cognitive functions supported by the default mode network, including autobiographical memory retrieval and future-oriented prospection, closely parallel the typical content of mind wandering (e.g. Philippi *et al.* 2015, Menon 2023, Jefferies and Smallwood 2025, Paquola *et al.* 2025). The ventral attention network, on the other hand, has been characterized as supporting stimulus-driven, bottom-up attentional reorienting (Corbetta *et al.* 2008), and more recently has been implicated in social reorienting processes (Hardikar *et al.* 2024). Therefore, the positive connectivity between these networks may reflect the connectivity patterns that support generating social scenarios, mentally simulating social interactions, and flexibly directing attention to interpersonal content.

Notably, both positive and negative rsFC network strengths associated with Social Cognition were associated with friendship quality (see Fig. 5). The positive association between positive rsFC network strength and friendship quality likely reflects effective coordination between internally oriented mentalizing processes subserved by the default mode network (Buckner *et al.* 2008, Andrews-Hanna *et al.* 2014) and stimulus-driven reorienting mechanisms supported by the ventral attention network (Corbetta *et al.* 2008). However, because these rsFC edges were identified based on their association with Social Cognition, which itself correlates with friendship quality, these findings are somewhat expected.

Two important implications emerge from the current findings. First, the identification of specific thought dimensions associated with friendship quality aligns with transdiagnostic perspectives (Christoff *et al.* 2016, Kucyi *et al.* 2023), suggesting that patterns of thought operate as core mechanisms that cut across diagnostic categories (Ehring 2021, Egan *et al.* 2025). While the present sample consisted of healthy adults, future work should examine how these thought dimensions are present in clinical populations (e.g. individuals with major depressive disorder), and whether interventions targeting thought content (e.g. cognitive-behavioral therapy) can produce measurable changes in both subjective thought patterns and neural network organization. Second, the findings that distinct ReHo clusters and rsFC patterns associated with different thought dimensions support an emerging view in cognitive neuroscience: the resting brain is organized in spatially and functionally heterogeneous patterns that reflect meaningful variations in subjective experience and dispositional tendencies (Yu *et al.* 2025).

There are several limitations that should be considered. First, although we used a data-driven approach well suited for identifying latent structure within heterogeneous self-report items (i.e. exploratory factor analysis) to decompose the NYC-Q (Gorgolewski *et al.* 2014), the resulting factor solution is inherently sample dependent. Accordingly, the present factors should not be interpreted as a close replication of prior NYC-Q or MDES structures. Second, all measures of thought content and adaptive functioning relied solely on self-report, which is susceptible to biases in self-presentation, insight, and recall (Podsakoff *et al.* 2003). Third, the study did not incorporate behavioral or observational indices of real-world social functioning, limiting inferences regarding how social-related thought relates to enacted social behavior. Fourth, the regression analysis linking rsFC network strengths to friendship quality does not constitute strict out-of-sample prediction, and cross-validation in independent samples is needed to evaluate the generalizability of these associations. Approaches such as lesion network mapping could potentially elucidate which specific nodes or connections within the identified networks are most critical to network integrity and dynamics (Alstott *et al.* 2009).

In conclusion, the current study shows that latent dimensions of thought during rest are associated with distinct patterns of ReHo and rsFC and with meaningful variations in social functioning, particularly friendship quality. In particular, social-related thought covaried with higher friendship quality, and this thought dimension was linked to robust local neural synchronization within mentalizing regions and connectivity between the default mode and ventral attention networks. The findings that both positive and negative rsFC network strengths related to Social Cognition were associated with friendship quality further suggest that social thought, social functioning, and large-scale connectivity patterns may be interrelated. Together, our results highlight the importance of integrating subjective phenomenological experience into cognitive neuroscience research. Future investigations employing longitudinal designs, clinical populations, and mechanistic interventions will further clarify how resting-state thought content shapes, and is shaped by, the development and maintenance of social relationships across the lifespan.

## Supplementary Material

nsag069_Supplementary_Data

## Data Availability

The data were sourced from a larger cross-sectional project (http://fcon_1000.projects.nitrc.org/indi/retro/MPI.html) conducted at the Max Planck Institute for Human Cognitive and Brain Sciences in Leipzig, Germany, and made available via the Neuroimaging Informatics Tools and Resources Collaboratory (https://www.nitrc.org/projects/mpilmbb/).
