# Supplementary material for: Social mind at rest: resting-state connectivity associated with social thought relates to friendship quality
Source: Soc Cogn Affect Neurosci. 2026 Aug 25;21(1):nsag069. doi: 10.1093/scan/nsag069 (PMC13581425; doi:10.1093/scan/nsag069)
Supplement: Supplementary file 1 [file nsag069_supplementary_data.docx]

**Supplementary material**

## **Magnetic resonance imaging (MRI) data acquisition**

The MRI data were recorded on a 3T scanner (MAGNETOM Verio, Siemens Healthcare, Erlangen, Germany) equipped with a 32-channel head coil. The high-resolution structural image was recorded using a 3D MP2RAGE sequence (Marques et al., 2010) with the following parameters: voxel size = 1.0 mm isotropic, field of view = 256 × 240 × 176 mm, repetition time (TR) = 5000 ms, echo time (TE) = 2.92 ms, first inversion time (TI_1_) = 700 ms, second inversion time (TI_2_) = 2500 ms, flip angle_1_ = 4°, flip angle_2_ = 5°, bandwidth = 240  Hz/Px, GRAPPA acceleration with iPAT factor 3 (32 reference lines), pre-scan normalization, duration = 8.22 min.

Four resting-state functional MRI (rs-fMRI) scans were obtained in axial orientation using T2*-weighted gradient-echo echo-planar imaging with multiband acceleration. The phase-encoding direction was A-P for runs 1 and 3, and P-A for runs 2 and 4. For all four runs, the parameters were set as follows: voxel size = 2.3 mm isotropic, imaging matrix = 88 × 88, 64 slices with 2.3 mm thickness, TR = 1400 ms, TE = 39.4 ms, flip angle = 69°, echo spacing = 0.67 ms, bandwidth = 1776 Hz/Px, partial fourier 7/8, multiband acceleration factor = 4, 657 volumes, duration = 15.30 min. To correct susceptibility-induced distortions in the fMRI data, additional spin-echo EPI images were acquired with matching parameters. During the rs-fMRI scans, participants were instructed to remain awake and lie still with their eyes open while looking at a fixation cross.

## **Resting-state functional MRI (rs-fMRI) data preprocessing**

The rs-fMRI volumes were preprocessed using fMRIPrep version 24.0.1 (Esteban et al., 2019) using default parameters. Briefly, functional data were slice-time corrected using 3dTshift from AFNI (Cox, 1996) and motion corrected using MCFLIRT (Jenkinson et al., 2002). This process was followed by co-registration to the corresponding T1w using boundary-based registration (Greve & Fischl, 2009) with nine degrees of freedom, using bbregister from FreeSurfer (Fischl, 2012). Motion correcting transformations, BOLD-to-T1w transformation, and T1w-to-template (MNI) warp were concatenated and applied in a single step using antsApplyTransforms (Avants et al., 2011) employing Lanczos interpolation.

Subsequently, the preprocessed volumes were denoised by regressing out six head motion parameters, global signal, and signals from white matter and cerebrospinal fluid, along with their temporal and quadratic derivatives, using XCP-D version 0.10.6 in ‘linc’ mode (Mehta et al., 2024). The denoised time series were then band-pass filtered using a second-order Butterworth filter to retain frequencies between 0.01 and 0.08 Hz. fMRI runs with excessive head motion were excluded from further analyses. For each participant, denoised time series data from the four scanning runs were concatenated into a single time series.

These concatenated time series data were parcellated into 200 cortical nodes using the Schaefer-200 atlas (Schaefer et al., 2018) and 19 subcortical nodes based on FreeSurfer subcortical segmentations (Fischl et al., 2002). For each participant, a 219 x 219 resting-state functional connectivity (rsFC) matrix was generated by computing pairwise Pearson correlations between nodes in the parcellated time series and applying Fisher’s *z* transformation. The rsFC matrices were then vectorized by extracting the lower triangular elements (excluding the diagonal) and reshaping them into a single column of values (i.e., rsFC vectors). These vectorized rsFC matrices were used as inputs for the subsequent rsFC analyses.

Resting-state regional homogeneity (ReHo) cortical surface images were derived from these denoised and band-pass-filtered volumes and mapped onto the FSLR32K surface template (i.e., 64,984 vertices) using XCP-D (Mehta et al., 2024). For each vertex, Kendall’s coefficient of concordance was calculated using the time series of its neighboring vertices based on 2D ReHo-1 (neighbor size = 1; Jiang & Zuo, 2016; Zang et al., 2004), yielding a vertex-wise ReHo measure across the cortex. The resulting ReHo images were then averaged across runs for each participant and smoothed using a 5 mm full-width at half-maximum Gaussian kernel.

Note that spatial smoothing was applied to the ReHo cortical surface images after ReHo computation but not to the functional connectivity data. This approach was used because smoothing ReHo maps can improve the signal-to-noise ratio and facilitate cluster formation for the vertex-wise statistical analyses without influencing the local synchronization estimates from which ReHo was derived (Jiang & Zuo, 2016). In contrast, rsFC analyses were conducted using parcellated time series from the Schaefer-200 atlas, for which signal averaging within functionally homogeneous parcels already provides a degree of spatial regularization and dimensionality reduction (Schaefer et al., 2018). Additional smoothing could introduce signal mixing across parcel boundaries and alter functional network estimates, thereby reducing the anatomical and network specificity of the rsFC estimates (Alakörkkö et al., 2017).

**References**

Alakörkkö, T., Saarimäki, H., Glerean, E., Saramäki, J., & Korhonen, O. (2017). Effects of spatial smoothing on functional brain networks. *European Journal of Neuroscience*, *46*(9), 2471-2480. <https://doi.org/10.1111/ejn.13717>

Avants, B. B., Tustison, N. J., Song, G., Cook, P. A., Klein, A., & Gee, J. C. (2011). A reproducible evaluation of ANTs similarity metric performance in brain image registration. *NeuroImage*, *54*(3), 2033-2044. <https://doi.org/10.1016/j.neuroimage.2010.09.025>

Cox, R. W. (1996). AFNI: Software for analysis and visualization of functional magnetic resonance neuroimages. *Computers and Biomedical Research*, *29*(3), 162-173. <https://doi.org/10.1006/cbmr.1996.0014>

Esteban, O., Markiewicz, C. J., Blair, R. W., Moodie, C. A., Isik, A. I., Erramuzpe, A., Kent, J. D., Goncalves, M., DuPre, E., Snyder, M., Oya, H., Ghosh, S. S., Wright, J., Durnez, J., Poldrack, R. A., & Gorgolewski, K. J. (2019). fMRIPrep: A robust preprocessing pipeline for functional MRI. *Nature Methods*, *16*(1), 111-116. <https://doi.org/10.1038/s41592-018-0235-4>

Fischl, B. (2012). FreeSurfer. *NeuroImage*, *62*(2), 774-781. <https://doi.org/10.1016/j.neuroimage.2012.01.021>

Fischl, B., Salat, D. H., Busa, E., Albert, M., Dieterich, M., Haselgrove, C., van der Kouwe, A., Killiany, R., Kennedy, D., Klaveness, S., Montillo, A., Makris, N., Rosen, B., & Dale, A. M. (2002). Whole brain segmentation: Automated labeling of neuroanatomical structures in the human brain. *Neuron*, *33*(3), 341-355. <https://doi.org/10.1016/S0896-6273(02)00569-X>

Greve, D. N., & Fischl, B. (2009). Accurate and robust brain image alignment using boundary-based registration. *NeuroImage*, *48*(1), 63-72. <https://doi.org/10.1016/j.neuroimage.2009.06.060>

Jenkinson, M., Bannister, P., Brady, M., & Smith, S. (2002). Improved optimization for the robust and accurate linear registration and motion correction of brain images. *NeuroImage*, *17*(2), 825-841. <https://doi.org/10.1016/s1053-8119(02)91132-8>

Jiang, L., & Zuo, X. N. (2016). Regional Homogeneity: A multimodal, multiscale neuroimaging marker of the human connectome. *The Neuroscientist*, *22*(5), 486-505. <https://doi.org/10.1177/1073858415595004>

Marques, J. P., Kober, T., Krueger, G., van der Zwaag, W., Van de Moortele, P.-F., & Gruetter, R. (2010). MP2RAGE, a self bias-field corrected sequence for improved segmentation and T1-mapping at high field. *NeuroImage*, *49*(2), 1271-1281. <https://doi.org/10.1016/j.neuroimage.2009.10.002>

Mehta, K., Salo, T., Madison, T. J., Adebimpe, A., Bassett, D. S., Bertolero, M., Cieslak, M., Covitz, S., Houghton, A., Keller, A. S., Lundquist, J. T., Luo, A., Miranda-Dominguez, O., Nelson, S. M., Shafiei, G., Shanmugan, S., Shinohara, R. T., Smyser, C. D., Sydnor, V. J.,…Satterthwaite, T. D. (2024). XCP-D: A robust pipeline for the post-processing of fMRI data. *Imaging Neuroscience*, *2*, imag-2-00257. <https://doi.org/10.1162/imag_a_00257>

Schaefer, A., Kong, R., Gordon, E. M., Laumann, T. O., Zuo, X.-N., Holmes, A. J., Eickhoff, S. B., & Yeo, B. T. T. (2018). Local-Global parcellation of the human cerebral cortex from intrinsic functional connectivity MRI. *Cerebral Cortex*, *28*(9), 3095-3114. <https://doi.org/10.1093/cercor/bhx179>

Zang, Y., Jiang, T., Lu, Y., He, Y., & Tian, L. (2004). Regional homogeneity approach to fMRI data analysis. *NeuroImage*, *22*(1), 394-400. <https://doi.org/10.1016/j.neuroimage.2003.12.030>
